# Non-consumptive effects stabilize herbivore control over multiple generations

**DOI:** 10.1371/journal.pone.0241870

**Published:** 2020-11-10

**Authors:** Kathryn S. Ingerslew, Deborah L. Finke

**Affiliations:** Division of Plant Sciences, University of Missouri, Columbia, MO, United States of America; University of Manitoba, CANADA

## Abstract

Understanding the factors that influence predator-prey dynamics requires an investigation of oscillations in predator and prey population sizes over time. However, empirical studies are often performed over one or fewer predator generations. This is particularly true for studies addressing the non-consumptive effects of predators on prey. In a previous study that lasted less than one predator generation, we demonstrated that two species of parasitoid wasps additively suppressed aphid populations through a combination of consumptive and non-consumptive effects. However, the non-consumptive effects of one wasp reduced the reproductive success of the other, suggesting that a longer-term experiment may have revealed antagonism between the wasps. The goal of our current study is to evaluate multi-generation consumptive and non-consumptive interactions between pea aphids (*Acyrthosiphon pisum*) and the wasps *Aphidius ervi* and *Aphidius colemani*. *Aphidius ervi* is a common natural enemy of pea aphids. *Aphidius colemani* is a non-consumptive enemy that does not consume pea aphids, but negatively affects pea aphid performance through behavioral disturbance. Large field cages were installed to monitor aphid abundance in response to the presence and absence of both species of wasp over four weeks (two parasitoid generations). We found that the non-consumptive enemy *A*. *colemani* initially controlled the pea aphid population, but control in the absence of parasitism was not sustainable over the long term. *Aphidius ervi* suppressed pea aphids through a combination of consumptive and non-consumptive effects. This suppression was more effective than that of *A*. *colemani*, but aphid abundance fluctuated over time. Suppression by *A*. *ervi* and *A*. *colemani* together was complementary, leading to the most effective and stable control of pea aphids. Therefore, promoting a diverse natural enemy community that contributes to pest control through consumptive and non-consumptive interactions may enhance the stability of herbivore population suppression over time.

## Introduction

Non-consumptive effects, also known as non-lethal effects or trait-mediated interactions, are changes in prey phenotype (e.g., behavior, morphology, or physiology) in response to the perceived threat of predation [1]. Non-consumptive effects can ultimately impact prey population size by altering prey fitness or migration [2, 3]. In general, non-consumptive effects are considered common and can produce direct and indirect effects on herbivores and plants that are as strong as and sometimes stronger than consumptive effects [4]. However, a review of non-consumptive studies involving arthropods found that over half of the studies were completed in under 24 hours and only one third lasted for more than one week [5]. Thus, our understanding of how non-consumptive effects influence predator-prey population dynamics is largely based on studies that are limited to a single predator and/or prey generation, and often less [5, 6].

Increasing the temporal scale of non-consumptive studies to accommodate reproduction provides insight into how non-consumptive effects may influence population cycles [7–9]. Studies of sufficient duration to include prey reproduction reveal carryover of non-consumptive effects on subsequent generations of prey [10–12]. For example, grasshoppers under chronic risk of predation alter their jumping mechanics to more quickly escape from spider predators [13]. However, the resulting offspring are smaller and cannot jump as far nor evade predators as effectively as the offspring of grasshoppers reared under no predation risk. Hence, traits that enhance survival in one generation may predispose the subsequent generation to higher predation risk. Some studies have explored legacy effects such as this for prey [14, 15], but the same is not true for predators. Predator reproduction is rarely incorporated into empirical non-consumptive studies. The common expectation based on consumption is that a decline in prey abundance will lead to an increase in predator abundance as predators eat prey and convert prey biomass into predator biomass through reproduction [16–19]. However, non-consumptive suppression of prey, which decouples prey suppression from predator reproduction, can provide alternative explanations for common predator-prey patterns documented in nature [7–9].

From the predator perspective, one of the constraints to conducting multi-generational studies is the challenge of establishing and sustaining relevant treatments. The common approach is to create treatments where prey are subjected to either a proxy of predator presence or a modified non-lethal predator that cannot consume prey. For example, prey may be exposed to odors associated with predation [e.g., 12, 20, 21] or subjected to simulated predator attack by means of poking or disturbing prey without the predator being present [e.g., 22]. Although these methods work well for studies focused on the short-term impact of predators on prey populations, neither of these approaches utilize an actual predator, and thus there is no opportunity to explore the impacts of non-consumptive effects on multi-generational predator and prey interactions. Another common technique is to modify predator mouthparts by gluing or clipping them, so that predators can hunt and attack, but are physically unable to consume prey [e.g., 23, 24]. In this case, prey interact with a living predator; however, the predator may not reproduce due to the inability to feed. Furthermore, if the predator does reproduce, the offspring will have fully-functioning mouthparts and be capable of feeding on prey, thus the treatment will not be maintained. Difficulty in teasing apart the role of consumption versus behavioral interactions while still allowing for predator reproduction, limits our ability to examine the multi-generational contribution of these interactions to predator and prey dynamics.

A unique opportunity to quantify non-consumptive effects without artificial manipulation arises when prey respond defensively to non-enemy organisms due to the erroneous perception of predation risk [25, 26]. The parasitoid wasp *Aphidius ervi* Haliday (Hymenoptera: Braconidae) is a common natural enemy of pea aphids (*Acyrthosiphon pisum* (Harris), Hemiptera: Aphididae) that reduces pea aphid abundance through a combination of consumptive and non-consumptive effects [27–29]. The closely-related wasp *Aphidius colemani* Viereck does not parasitize pea aphids [30], but still contributes to pea aphid suppression through non-consumptive behavioral interactions such as provoking the pea aphid escape response of dropping from a host plant [26, 31]. In a study that encompassed less than one parasitoid generation, we found that the natural enemy *A*. *ervi* and the non-enemy *A*. *colemani* additively suppressed the pea aphid population when present together, but a decline in the number of *A*. *ervi* pupae (mummies) that formed in the presence of *A*. *colemani* suggested the potential for long-term interference [29]. *Aphidius ervi* oviposition success declines when abiotic conditions enhance the pea aphid drop-off response [32], and the same may be true in response to contact with *A*. *colemani*. The short-term nature of the experiment did not allow for an investigation of the potential for interference to influence the size of future generations of *A*. *ervi* wasps or the magnitude and stability of pea aphid suppression over time.

Here we explore how the addition of a non-consumptive enemy influences the interaction between a parasitoid natural enemy and its host over multiple generations. Based on our previous results, we hypothesize that the non-consumptive enemy *A*. *colemani* will reduce the reproductive potential of the natural enemy *A*. *ervi*. Thus, non-consumptive effects are predicted to interfere with parasitoid-host dynamics, weakening pea aphid suppression and reducing plant productivity over the long term.

## Methods

We factorially manipulated the presence of *A*. *colemani* and *A*. *ervi* in large field cages and monitored the pea aphid population over four weeks, or two parasitoid generations. The study was conducted at Bradford Research Farm (Columbia, MO) from 17-June-2014 until 24-July-2014. This is a university-owned research farm. No permits were required, and studies did not involve endangered or protected species. Experimental units were 2 m x 2 m x 2 m field cages that were buried approximately 30 cm into the soil to deter any organisms from entering the cages. Inside each cage, we transplanted three rows of four eight-day-old fava bean plants (*Vicia faba* L.). After 7 d, 10 pea aphids were released on each fava bean plant. Each cage also contained three rows of four 40-day-old collard plants (*Brassica oleracea* L.), with 10 green peach aphids (*Myzus persicae* (Sulzer)) per plant. *Aphidius colemani* wasps do not parasitize pea aphids, thus green peach aphids and their host plants were included to maintain the *A*. *colemani* population throughout the study. Green peach aphids were not the primary focus of this study, but data regarding green peach aphid populations are included in supplementary material (S1 Appendix).

After a 24-h settling period for the aphids, one of four treatments was randomly assigned to each cage: (1) control with no parasitoids present, (2) 30 adult *A*. *ervi*, (3) 30 adult *A*. *colemani*, or (4) a mixed treatment of 30 adult *A*. *ervi* and 30 adult *A*. *colemani*. Parasitoids were released at a sex ratio of 1:1. Treatments were replicated seven times. After the initial release of parasitoid wasps, pea aphid abundance and the number of pea aphid mummies were counted every 7 d over 4 wk for a total of four sampling periods. After the final count, the aboveground biomass of the pea aphids’ fava bean host plants was harvested, dried, and weighed.

Aphids used in this study were reared in cages in the Ashland Road Greenhouse facility on the campus of the University of Missouri (Columbia, MO, 16:8 L:D, 26–38°C) for many generations prior to use. Pea aphids were originally collected from an alfalfa field (*Medicago sativa* L.) and were maintained on fava bean plants. Green peach aphids were collected from and reared on greenhouse collard plants. *Aphidius ervi* and *A*. *colemani* wasps were purchased from Rincon-Vitova Insectaries (Ventura, California).

The main and interactive effects of the presence of *A*. *ervi* and *A*. *colemani* on the size of the pea aphid population over time were analyzed with a repeated-measures analysis of variance (ANOVA) (Proc Mixed, SAS v.9.4, SAS Institute, Cary, NC). The variance-covariance structures tested were: compound symmetry, heterogeneous compound symmetry, first-order autoregressive, heterogeneous first-order autoregressive, Toeplitz, and unstructured. First-order autoregressive variance-covariance structure was determined as the best fit according to the lowest AIC value.

Temporal stability in pea aphid suppression was calculated using the coefficient of variation (standard deviation / mean) of pea aphid population size in each experimental unit over time. The main and interactive effects of the presence of *A*. *ervi* and *A*. *colemani* on the coefficient of variation of pea aphid abundance were analyzed using a two-way ANOVA. During the study, pea aphid populations grew to such a large size in the control treatment and the treatment where *A*. *colemani* was present alone that they over-exploited the fava bean plants and led to plant death. Plant death led to a dramatic decline in the pea aphid population size in subsequent sampling dates in these two treatments. To account for this, we also compared the coefficient of variation in pea aphid abundance between the two treatments where pea aphid populations did not overexploit the fava bean plants: where *A*. *ervi* was present alone and where both *A*. *ervi* and *A*. *colemani* were present.

The cumulative abundance of pea aphid mummies was compared between the treatment where *A*. *ervi* was alone and the mixed treatment with both *A*. *ervi* and *A*. *colemani* present with a one-way ANOVA. The analysis only included two treatments because pea aphid mummies only formed in treatments where *A*. *ervi* was present. *A*. *colemani* cannot parasitize pea aphids and its effect is entirely non-consumptive [33].

To assess the indirect effect of wasps on plants, the main and interactive effects of *A*. *colemani* and *A*. *ervi* presence on dried aboveground biomass of fava bean plants at the conclusion of the experiment were analyzed using a two-way ANOVA.

All variables were log-transformed to adhere to the assumptions of an ANOVA. Due to human error on the second count day (July 10), three values for pea aphid abundance from the control treatments were not included in the analyses.

## Results

**Fig 1:**
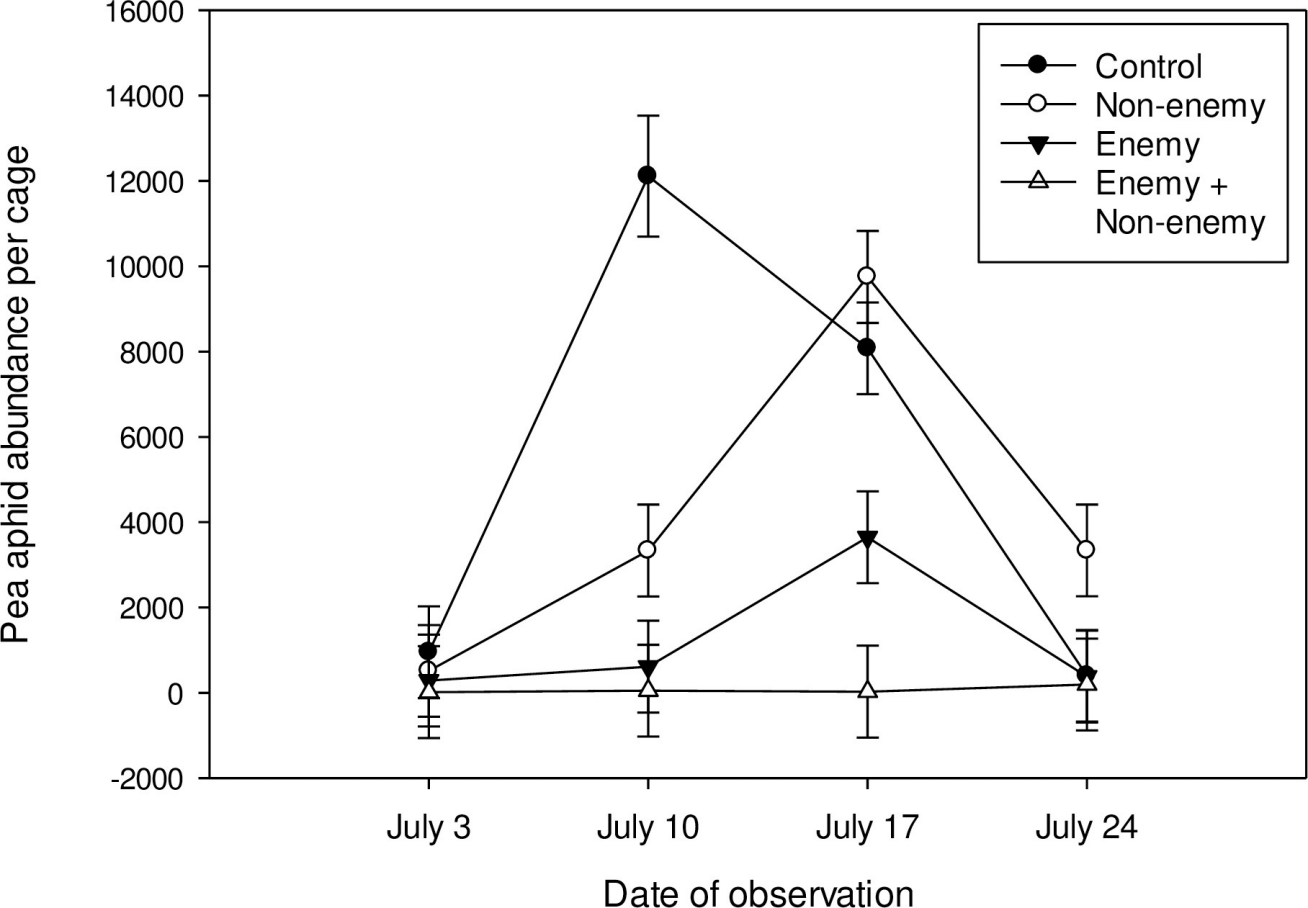
Impact of the presence of the natural enemy *Aphidius ervi* and non-consumptive enemy *Aphidius colemani* on the abundance of pea aphid (*Acyrthosiphon pisum*) populations over four weeks, two parasitoid generations. *Aphidius ervi* and *A*. *colemani* were released into the cages on June 26, one week prior to the first date of observation. LS means ± 1 SEM of untransformed data are shown.

There was a three-way interaction between the presence of *A*. *colemani*, *A*. *ervi*, and the day of observation on pea aphid abundance (*F*_3,68.5_ = 4.31, *P* = 0.0076, Fig 1), indicating that the interactions between the natural enemy and non-consumptive enemy wasps were not consistent over the course of the study. In the first two weeks of the study (July 3 and July 10), suppression by the two wasp species was additive (enemy*non-enemy interaction, July 3: *F*_1,24_ = 0.21, *P* = 0.6512, July 10: *F*_1,21_ = 1.00, *P* = 0.3291). In the last two weeks, suppression was synergistic (enemy*non-enemy interaction, July 17: *F*_1,24_ = 8.70, *P* = 0.0070, July 24: *F*_1,24_ = 7.15, *P* = 0.0135). However, this statistical interaction is likely not biologically relevant since pea aphids overexploited the host plants in the control treatment and the bottom-up effect of reduced plant quality led to a dramatic decline in the pea aphid population size. The natural enemy *A*. *ervi* reduced pea aphid population size (*F*_1,33.9_ = 61.62, *P* < 0.0001), but suppression was not consistent over the course of the study, with a spike in pea aphid abundance on the third date (enemy*date interaction: *F*_3,68.5_ = 8.76, *P* < 0.0001). The non-consumptive enemy *A*. *colemani* also did not exert consistent suppression on the pea aphid population over time (non-enemy*date interaction: *F*_3,68.5_ = 6.18, *P* = 0.0009) and had no main effect on pea aphid population size (*F*_1,33.9_ = 1.75, *P* = 0.1947). There was no evidence of an indirect interaction between *A*. *ervi* and *A*. *colemani* mediated by the presence of green peach aphids (the host for *A*. *colemani*) (S1 Appendix). *A*. *ervi* did parasitize green peach aphids at low levels in some cages. However, the rate of parasitism was not enough to suppress green peach aphid abundance relative to the no-wasp control. Furthermore, there was no interaction between the presence of *A*. *ervi* and *A*. *colemani* on the number of green peach aphid mummies formed.

When all four treatments were included in the analysis, including those where the aphids overexploited the plants, there was a main effect of the presence of the non-enemy *A*. *colemani* on the coefficient of variation of pea aphid abundance (*F*_1,21_ = 9.64, *P* = 0.0054; main effect mean ± SE: 1.38 ± 0.11 in the absence of *A*. *colemani*, 0.97 ± 0.09 in the presence of *A*. *colemani*), indicating that the presence of the non-consumptive enemy reduced the variability in the abundance of pea aphids over time, i.e., led to a more stable pea aphid population size. There was no main effect of the natural enemy *A*. *ervi* or an interaction between the enemy and the non-enemy on the stability of the pea aphid population size (*F*_1, 21_ = 3.00, *P* = 0.0981; *F*_1, 21_ = 1.65, *P* = 0.2135, respectively). However, when the two treatments where the aphid populations grew to such a large level that they killed their host plants were removed from the analysis, the coefficient of variation in pea aphid abundance was lower in the mixed treatment where both species of parasitoid were present, compared to when the enemy *A*. *ervi* was present alone (*t*_12_ = 2.50, *P* = 0.0278; mean ± SE: 1.34 ± 0.14 in the absence of *A*. *colemani*, 0.83 ± 0.14 in the presence of *A*. *colemani*). The addition of the non-consumptive enemy *A*. *colemani* reduced the variability and increased the stability in pea aphid population size over time.

**Fig 2:**
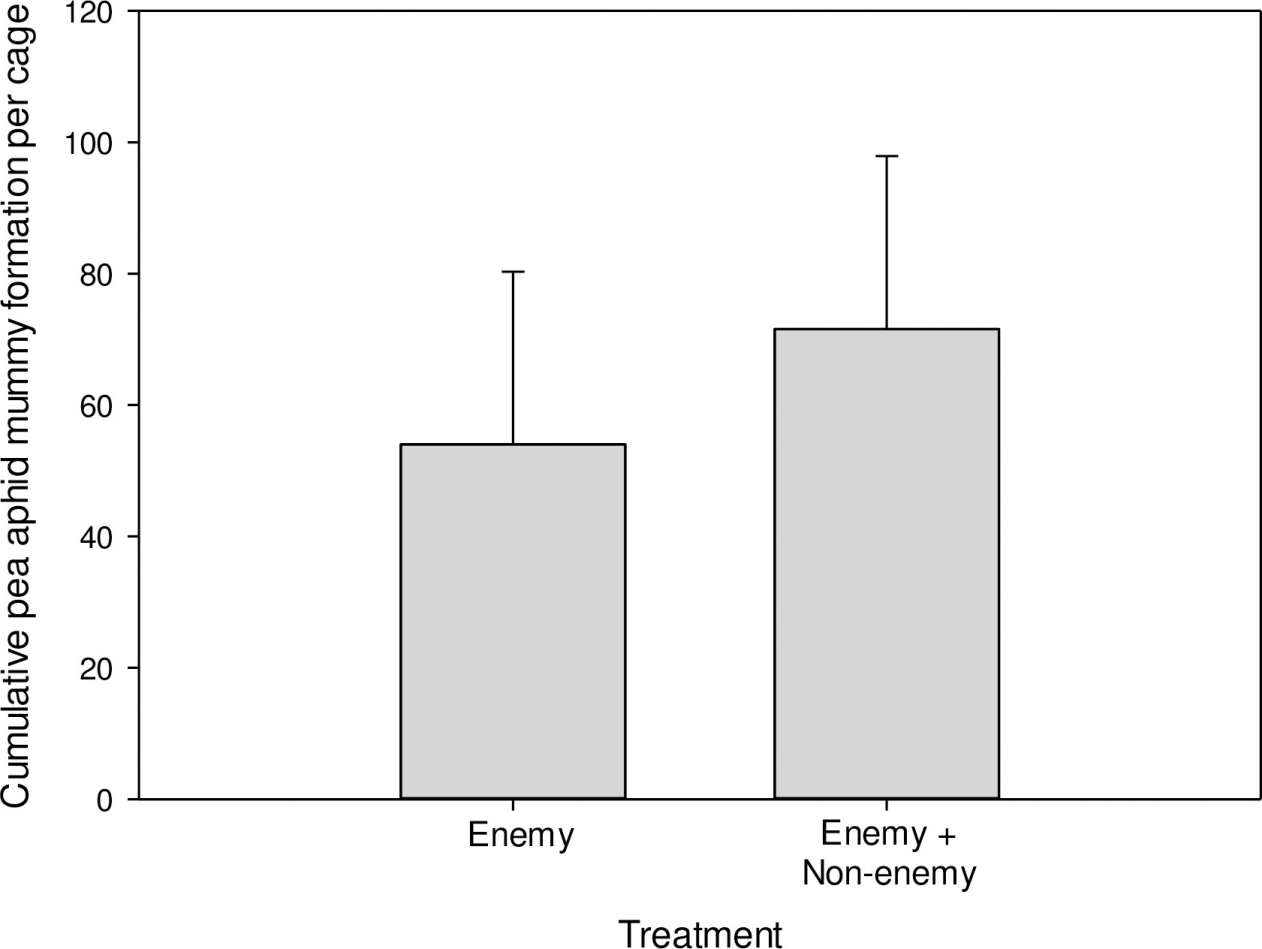
Cumulative pea aphid (*Acyrthosiphon pisum*) mummy formation over the course of the experiment in response to the presence of the natural enemy *Aphidius ervi* and non-consumptive enemy *Aphidius colemani*. *Aphidius ervi* is the only parasitoid that parasitizes pea aphids and forms mummies, so only treatments where *A*. *ervi* was present are shown. LS means ± 1 SEM of untransformed data are shown.

The presence of the non-consumptive enemy *A*. *colemani* did not interfere with reproduction by *A*. *ervi*. There was no difference in the total pea aphid mummy formation by *A*. *ervi* when *A*. *ervi* was alone or in the mixed treatment with *A*. *colemani* (*t*_12_ = 0.44, *P* = 0.6700, Fig 2).

**Fig 3:**
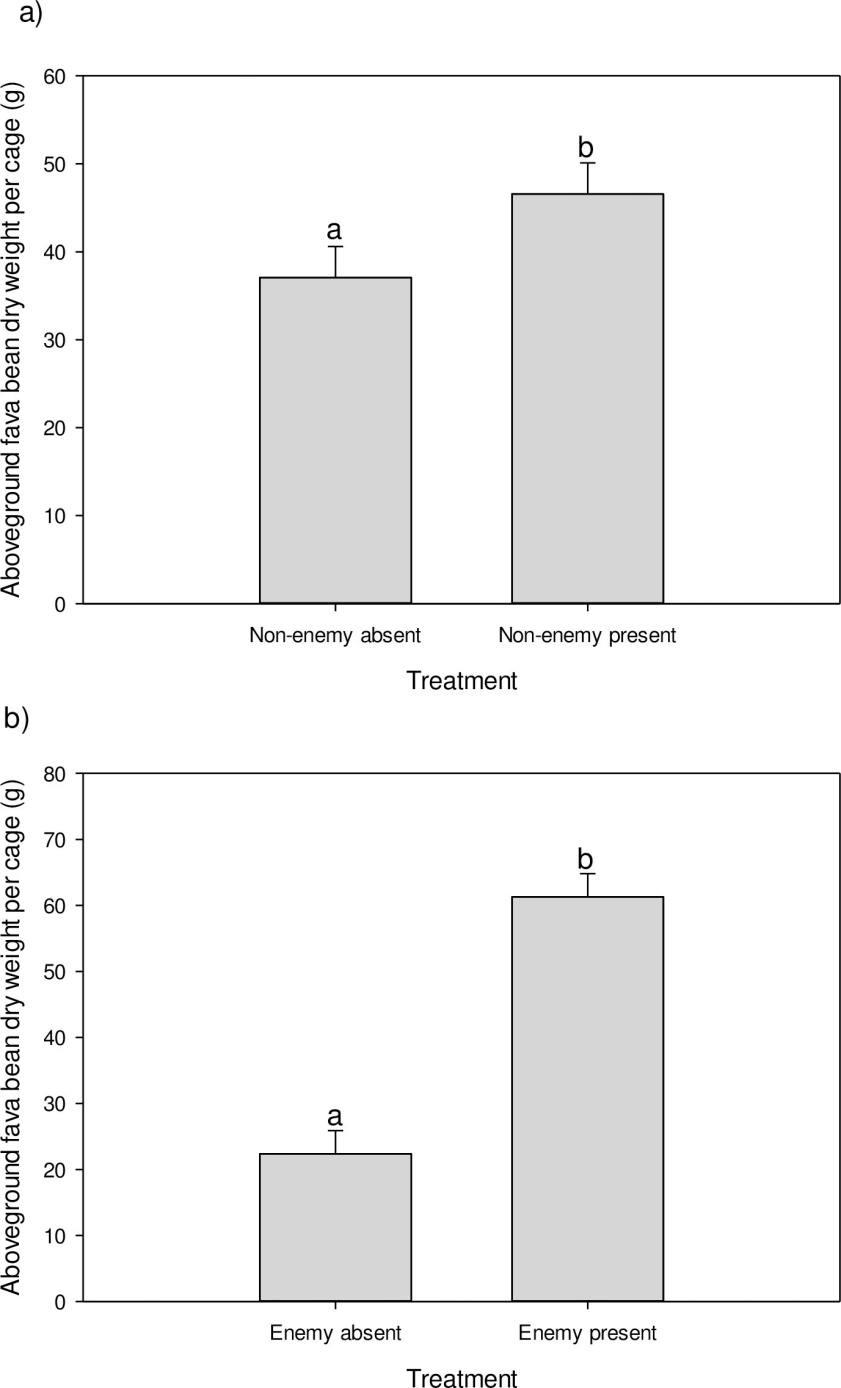
Impact of the presence of the natural enemy *Aphidius ervi* and non-consumptive enemy *Aphidius colemani* on dried aboveground fava bean (*Vicia faba*) plant biomass at the completion of the experiment. (a) Main effect of the non-consumptive enemy *A*. *colemani*. (b) Main effect of the natural enemy *A*. *ervi*. LS means ± 1 SEM of untransformed data are shown. Means with different letters are significantly different at the α < 0.05 level.

There were main effects of the presence of *A*. *colemani* and *A*. *ervi* on dried aboveground fava bean plant biomass (*A*. *colemani*: *F*_1, 24_ = 4.34, *P* = 0.0480, Fig 3A; *A*. *ervi*: *F*_1, 24_ = 55.90, *P* < 0.0001, Fig 3B). The addition of either *A*. *colemani* or *A*. *ervi* led to greater dried aboveground plant biomass of fava bean plants. There was no interaction in the effects of the presence of *A*. *colemani* and *A*. *ervi* on the dried aboveground fava bean plant biomass (*F*_1, 24_ = 0.00, *P* = 0.9788).

## Discussion

Non-consumptive suppression of prey by predators is well documented, but our understanding of the importance of non-consumptive effects for predator-prey interactions is limited by the short duration of most experimental studies [5]. This is partly due to the logistical difficulty of creating treatments that tease apart non-consumptive from consumptive effects in a manner that will persist across predator generations. Taking advantage of the defensive behavioral response of pea aphids to non-enemy parasitoid wasps, we were able to quantify the non-consumptive effects of wasps on aphids over multiple generations. Consistent with previous short term studies [26], we found that non-consumptive effects alone were sufficient to initially suppress pea aphid abundance where only the non-enemy *A*. *colemani* was present. However, suppression was transient in the absence of consumptive effects and aphids escaped control over the long term. Contrary to our prediction, a non-consumptive enemy did not disrupt parasitoid-host interactions [29]. Alternatively, the combination of consumptive and non-consumptive effects stabilized pea aphid suppression, yielding the lowest and least variable pea aphid population size over the long term.

The ability of natural enemies, including predators and parasitoids, to reduce aphid population growth by stimulating defense behaviors is well documented [24, 34]. More recent evidence suggests that aphid performance is also negatively affected by disturbance from non-enemy parasitoids and other non-predaceous, non-competitive commensal species [25, 26, 29]. For example, fruit flies negatively affect bird cherry-oat aphid (*Rhopalosiphum padi*) population growth because the stress response of aphids does not discriminate between enemies and flies in search of food [25]. In agreement with these previous short-term studies, we found reduced pea aphid abundance in the presence of the non-enemy wasp *A*. *colemani*, but only in the first two weeks of the study. Over the next two weeks, the pea aphid population eventually grew so large that the fava bean host plants were over-exploited and died. Pea aphids likely escaped control over the long term because *A*. *colemani* population growth is not coupled with that of non-prey pea aphids as predicted by consumptive predator-prey models [16–19]. *Aphidius colemani* wasps persisted in the system for the duration of the study, due to the presence of their green peach aphid prey on collard plants (S1 Appendix). Therefore, while many short-term studies find that non-consumptive effects are strong and prevalent, longer term studies are necessary to understand whether and how the impacts of these interactions will scale up.

*Aphidius ervi* is a relatively specialized natural enemy of pea aphids with populations that are often coupled to that of their hosts [35, 36]. Accordingly, we found that *A*. *ervi* alone consistently maintained pea aphid populations at levels well below the no-wasp control. As in other studies [37], we found that the magnitude of pea aphid suppression by *A*. *ervi* fluctuated over time with an increase in aphid abundance in the third week. Wasp preference for particular aphid developmental stages and variation in host preferences across individual wasp females or females of different ages have been invoked as mechanisms to explain similar patterns in the past [38]. Although, some have questioned the importance of such individual behaviors in influencing overall aphid-parasitoid dynamics [39].

Contrary to our prediction, the addition of the non-consumptive enemy *A*. *colemani* did not disrupt control of pea aphids by *A*. *ervi*. We previously documented a reduction in *A*. *ervi* reproduction in the presence of *A*. *colemani*, which was attributed to behavioral interference [29]. We found no evidence of such interference in this longer-term study. Rather, the effects of the non-consumptive enemy and the natural enemy were complementary, with the addition of *A*. *colemani* preventing the temporary spike in aphid abundance at week 3 and reducing the variability of pea aphid population control over time. We attribute this response to temporal niche partitioning due to differences in development time of the two parasitoid species. *Aphidius colemani* develops, on average, two days faster than *A*. *ervi* [40, 41]. As a result, adults of the non-consumptive enemy *A*. *colemani* were actively foraging in the environment and maintaining suppression of pea aphid abundance through behavioral interactions [26, 31] during times when *A*. *ervi* was inactive and in the pupal stage. Therefore, the presence of the non-consumptive enemy *A*. *colemani* may provide insurance against pea aphid outbreaks at times when the consumptive enemy, *A*. *ervi*, is inactive or present at low densities. Many mechanisms have been explored to explain the stability of parasitoid and host interactions, including foraging behavior, spatial processes, and mutual interference [37, 42–45]. Our study demonstrates that behavioral interactions in the form of complementary consumptive and non-consumptive suppression by parasitoid wasps may also lead to increased stability of host populations over time.

Previous studies have shown that non-consumptive interactions not only affect herbivore prey, but can also cascade down to indirectly impact plants [4, 46, 47]. In our study, the pea aphid natural enemy *A*. *ervi* controlled pea aphid populations, resulting in increased aboveground biomass of the fava bean plants. The addition of the non-consumptive enemy *A*. *colemani* led to more consistent suppression of pea aphid populations than when *A*. *ervi* was alone. However, the greater control of the pea aphid population was not reflected in an increase in fava bean plant biomass, although there was a trend for the highest plant biomass to be achieved when both species of wasp were present. Interestingly, the presence of the non-consumptive enemy alone also increased fava bean biomass. Thus, despite the short-term nature of pea aphid suppression by the non-consumptive enemy, suppression was still sufficient to benefit fava bean plants.

Our study demonstrates the importance of spatial and temporal scale in influencing the outcome of ecological interactions [48–50]. In a previous study done in small cages over one parasitoid generation, we saw evidence of potential antagonism between these two species of wasps and their combined consumptive and non-consumptive effects on pea aphid population size [29]. However, in this multi-generation study, we found the greatest pea aphid suppression when both species were present. In addition, we document a novel role for non-consumptive enemy species in the environment: to prevent outbreaks of herbivore populations at times when consumer densities are low [51, 52]. A species that would otherwise not be included in a food web or trophic interaction contributed to enhanced herbivore suppression and increased plant productivity through non-consumptive mechanisms.

## Supporting information

S1 Appendix Green peach aphid population responses. (DOCX)

S1 Material (XLSX)
