# Supplementary material for: Non-consumptive effects stabilize herbivore control over multiple generations
Source: PLoS One. 2020 Nov 10;15(11):e0241870. doi: 10.1371/journal.pone.0241870 (PMC7654827; doi:10.1371/journal.pone.0241870)
Supplement: S1 Appendix — (DOCX) [file pone.0241870.s001.docx]

**S1 Appendix.** **Green peach aphid population responses.**

**Statistical methods:**

The main and interactive effects of the presence of *A. ervi* and *A. colemani* on the size of the green peach aphid population over time was analyzed with a repeated-measures analysis of variance (ANOVA) (Proc Mixed, SAS v.9.3, SAS Institute, Cary, NC). The most appropriate variance-covariance structure was chosen according to the lowest AIC value. The variance-covariance structures that were tested were: compound symmetry, heterogeneous compound symmetry, first-order autoregressive, heterogeneous first-order autoregressive, Toeplitz, and unstructured. First-order autoregressive variance-covariance structure was determined as the best fit for the green peach aphid population size. Green peach aphid abundance was log-transformed to adhere to the assumptions of an ANOVA.

The variability in green peach aphid abundance over time (i.e., temporal stability) was calculated using the coefficient of variation (standard deviation / mean) of green peach aphid population size in each experimental unit over time. The main and interactive effects of the presence of *A. ervi* and *A. colemani* on temporal stability was analyzed using a two-way ANOVA.

A generalized linear model assuming a log-normal probability distribution was used to analyze the main and interactive effects of the presence of *A. colemani* and *A. ervi* on the cumulative number of green peach aphid mummies that formed over the duration of the experiment.

The main and interactive effects of *A. colemani* and *A. ervi* presence on dried aboveground biomass of collard plants (the host plant of green peach aphids) at the conclusion of the experiment were analyzed using a two-way ANOVA. One sample of aboveground collard biomass mysteriously disappeared.

**Results:**

Parasitoid treatments did not affect green peach aphid abundance. Date of observation was the only significant effect of any predictive variable in the repeated-measures ANOVA on green peach aphid abundance (*F*_3, 61.7_ = 251.43, *P* < 0.0001, Fig. S1), indicating that green peach aphid abundance increased steadily over time in all treatments. There was no interaction between *A. colemani* and *A. ervi* and no main effects of either parasitoid species on the stability of green peach aphid populations over time, as measured by the coefficient of variation (*F*_1,24_ = 1.19, *P* = 0.2864; *F*_1,24_ = 0.50, *P* = 0.4878; *F*_1,24_ = 0.02, *P* = 0.8908, respectively).

There was no interaction in the effects of *A. ervi* and *A. colemani* on the total number of green peach aphid mummies that formed (*F*_1, 71_ = 1.84, *P* = 0.18, Fig. S2). As expected, the presence of *A. colemani* resulted in more green peach aphid mummies (*F*_1, 71_ = 5.29, *P* = 0.02).

There were no interactive or main effects of *A. colemani* and *A. ervi* on the dried aboveground plant biomass of the collard plants (*F*_1, 23_ = 2.19, *P* = 0.1528, *F*_1, 23_ = 1.48, *P* = 0.2363, and *F*_1, 23_ = 0.01, *P* = 0.9113, Fig. S3).

**Figure S1.** Impact of the presence of the parasitoid wasps *Aphidius ervi* Haliday and *Aphidius colemani* Viereck on green peach aphid, *Myzus persicae* (Sulzer), abundance over four weeks, approximately two parasitoid generations. LS means ± 1 SEM of untransformed data are shown.

**Figure S2.** Cumulative green peach aphid, *Myzus persicae* (Sulzer), mummy formation over the course of the experiment in response to the presence of *Aphidius ervi* Haliday and *Aphidius colemani* Viereck. LS means ± 1 SEM are shown. Means with different letters are significantly different at the α < 0.05 level.

**Figure S3.** Impact of the presence of the parasitoid wasps *Aphidius ervi* Haliday and *Aphidius colemani* Viereck on dried aboveground collard plant, *Brassica oleracea* L., biomass at the completion of the experiment. LS means ± 1 SEM are shown.
